# Physicochemical Properties of *Anopheles* Mosquito Larval Habitats in Nouakchott, Mauritania

**DOI:** 10.3390/tropicalmed11020042

**Published:** 2026-02-03

**Authors:** Mohamed Haidy Massa, Osman Abdillahi Guedi, Nicolas Gomez, Ali Ould Mohamed Salem Boukhary, Sébastien Briolant, Mohamed Aly Ould Lemrabott

**Affiliations:** 1 Unité de Recherche Génomes et Milieux (GEMI), Université de Nouakchott, Nouveau Campus Universitaire, Nouakchott BP 5026, Mauritania; medhaidy@gmail.com (M.H.M.); alimedsalem@gmail.com (A.O.M.S.B.); mohamedalylemrabott@yahoo.fr (M.A.O.L.); 2 Centre de Recherche en Sciences Humaines, Sociales, Langues et Littérature, Université de Djibouti, Campus de Balbala, Croisement RN2-RN5, Djibouti 77101, Djibouti; osman.guedi@gmail.com; 3 Département de Géographie, Université de La Réunion, 97400 Saint-Denis, France; 4 Unité Parasitologie et Entomologie, Département Risques Vectoriels, Institut de Recherche Biomédicale des Armées (IRBA), 13005 Marseille, France; nico13dna@hotmail.com; 5 Unité Mixte de Recheche Risques Infectieux Tropicaux et Microorganismes Emergents, Assistante Publique des Hopitaux de Marseille, Service de Santé des Armées, Aix Marseille University, 13005 Marseille, France; 6 IHU Méditerranée Infection, 13005 Marseille, France

**Keywords:** *Anopheles*, *An. arabiensis*, malaria, breeding sites, larvae, Nouakchott, Mauritania

## Abstract

Malaria remains one of the main public health problems in Mauritania, and it is essential to identify the factors that determine the distribution and productivity of *Anopheles* breeding sites in order to develop more effective control strategies. A longitudinal survey with repeated measurements was conducted in Nouakchott between May 2023 and April 2024, in order to examine the factors influencing the distribution and productivity of *Anopheles* larval habitats. The larvae were collected by immersion in 60 water points, once a month during the dry season and twice a month during the rainy season, for a total of 294 observations. The physical and chemical characteristics of the sites were also measured. Logistic regression analyses with random effects showed that the presence of *Culex* and *Aedes* larvae, pH, and temperature were statistically significantly associated with positive water collection for *Anopheles* larvae (aOR = 3.03, 95%CI [1.14–8.07], *p*-value = 0.026; aOR = 0.18, 95%CI [0.05–0.60], *p*-value = 0.006; aOR = 3.17, 95%CI [1.32–7.61], *p*-value = 0.010 and aOR = 5.95, 95%CI [2.09–16.92], *p*-value < 0.001, respectively). Only *Anopheles multicolor* and *An. arabiensis* were present in Nouakchott. Our results could help health authorities by guiding the destruction of breeding sites with biological larvicides or physical elimination of peridomestic habitats.

## 1. Introduction

The *Anopheles* mosquito is a vector for several diseases around the world, including malaria caused by *Plasmodium* spp. and lymphatic filariasis, the latter caused by the parasites *Wuchereria bancrofti* and *Brugia malayi*. It is also known to transmit O’nyong-nyong virus [1]. Malaria remains a significant public health challenge in 83 countries worldwide, with most deaths occurring in Africa. According to the Global Malaria Report, there were 263 million malaria cases and 597,000 malaria-related deaths in 2023 [2].

In Mauritania, malaria remains one of the major public health problems [3]. Transmission is intense and seasonal in the southern Sahel region, but unstable and sporadic in the northern Sahara region [4,5]. In this region of the Sahara, most cases of malaria are concentrated in Nouakchott [6,7]. Due to the short rainy season (July–September), malaria transmission peaks in October and November, following the rainy season [4]. The primary malaria vector in Nouakchott is *Anopheles arabiensis* [8]. *Plasmodium vivax* predominates, accounting for over 74% of PCR-confirmed cases, with *P. falciparum* and mixed infections making up smaller proportions [9].

Currently, pyrethroids are the only insecticides available for treating mosquito nets. However, due to widespread resistance to these compounds in Africa, these methods are gradually losing their effectiveness [10,11,12]. Furthermore, a recent study conducted in Nouakchott examined the resistance of *Aedes aegypti* to insecticides, revealing that the species is resistant to all those that were evaluated. [13] This is particularly the case with pyrethroids. Similar resistance is likely to be present in *Anopheles* mosquitoes. A study conducted on *Anopheles* populations sampled in Nouakchott between 2015 and 2016 demonstrated their resistance to pyrethroids [14]. In this situation, it is important to identify and characterize the breeding sites of *Anopheles* mosquitoes in order to develop effective strategies for the malaria control program [15]. Urbanization generally has a significant impact on the composition of the vector system and the dynamics of malaria transmission [16]. Some mosquito species have adapted to urban environments and prefer to lay their eggs in wastewater. This renders vector control efforts ineffective [17].

Larval habitats are determined by a series of physicochemical parameters, such as water temperature, pH, salinity, turbidity, conductivity, etc. Each mosquito species has its own preferences for larval breeding sites and fluctuations in these parameters affect vector development, influencing the abundance and morphology of adult mosquitoes [18,19,20,21,22]. Several studies have characterized the breeding sites of *Anopheles* mosquitoes across sub-Saharan Africa [23,24,25]. In Nouakchott, the first study of this type described the characteristics of the larval habitats of *An. gambiae s.l*. and showed that, despite the arid and dry climate of the Sahara, certain human practices, such as the construction of domestic water reservoirs or retention basins from public standpipes, promote the proliferation of mosquitoes and thus contribute to maintaining malaria transmission in the city [26].

A recent six-year retrospective study conducted in Nouakchott reported a significant decline in malaria prevalence among febrile patients, from 29.2% in 2015 to only 2.1% in 2020, reflecting the impact of interventions and environmental changes, such as reduced rainfall and improved drinking water systems [9]. However, few studies have been conducted on the physical and chemical characteristics of *Anopheles* breeding sites in Nouakchott. In addition, these studies were conducted over a very short period of time and on a limited number of parameters and breeding sites. The statistical analyses were also mainly descriptives.

This study provides comprehensive longitudinal monitoring over one year, enabling exhaustive seasonal analysis and detailed, integrated physicochemical profiling using a multiparametric probe. It uses robust mixed-effects models to analyze the co-occurrence of *Anopheles*, *Aedes*, and *Culex* species in a Saharan urban context.

## 2. Materials and Methods

### 2.1. Study Site

The study was conducted in Nouakchott, the capital of Mauritania, one of the largest cities in the Saharan region. Nouakchott is densely populated with approximately 1,500,000 inhabitants [27], and is located in the Atlantic coastal zone, characterized by low elevation between 1 m and 10 m below sea level. A belt of natural salt 1 to 2 km wide separates the city itself from the Atlantic coast.

The climate is characterized as Saharan, with low annual rainfall (<100 mm on average) and average annual temperatures and humidity of 27 °C and 56.5% respectively. Depending on the season, winds blow in several directions, with northern winds prevailing from October to May and westerly winds from June to September. [28]. The climate in Nouakchott is characterized by a long dry season from October to June and a short rainy season from July to September.

During the study period, the average maximum temperature was 33 °C, the average minimum temperature was 20.6 °C, and the average annual temperature ranged from 21.8 °C to 31.9 °C. The average annual relative humidity ranged from 28% to 69%. Precipitation was generally concentrated during the summer months (July, August and September), with an average of 120 mm per year. During the period studied, total precipitation reached 109.5 mm, distributed as follows: 30 mm in June, 15.5 mm in July, 54 mm in August, and 10 mm in September.

### 2.2. Study Period

**Figure 1:**
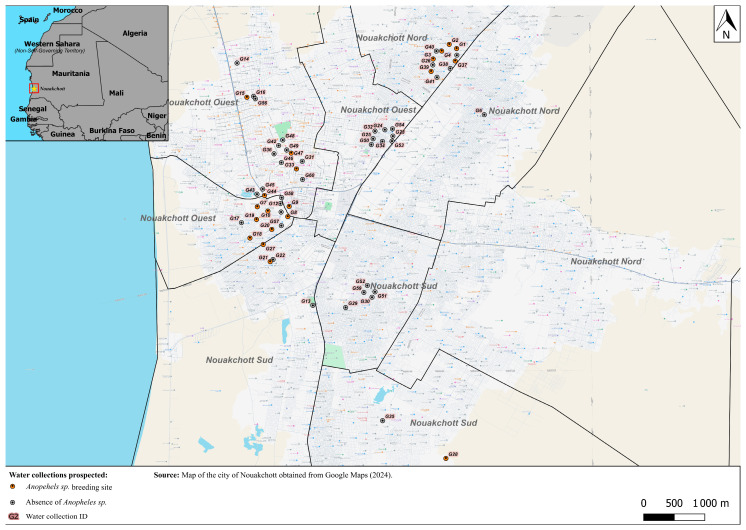
Map of water collection sites prospected in Nouakchott obtained from Google Maps (2024). The red circles indicate the *Anopheles* spp. breeding sites.

From May 2023 to April 2024, a longitudinal survey with repeated measurements was conducted in domestic and peridomestic environments to characterize mosquito breeding sites. A total of 60 water sources and containers were sampled (Figure 1), once per month during the dry season and twice per month during the rainy season as previously described in [13]. The collection sites were selected using a stratified approach in order to cover various urban contexts (residential areas of different socioeconomic levels, as well as peri-urban agricultural areas) to ensure maximum spatial and environmental representations of the city of Nouakchott.

All types of larval habitats observed during field surveys were included in the study, whether artificial or natural, permanent or temporary, and regardless of their accessibility, in order to minimize selection bias. The total number of water samples was deliberately limited to 60, as this number was considered sufficient to adequately reflect the diversity and distribution of water collections present at the urban scale.

### 2.3. Larval Collection and Morphological Identification of Adult Specimens

Different types of water-holding containers were examined as potential habitats for larvae, and mosquito larvae were sampled using the standard dipping with a mosquito dipper (BioQuip, Gardena, CA, USA) [29]. The larvae and pupae were collected using a pipette, or by emptying the containers completely when the larvae were small. All the larvae were counted to assess their density according to Papierok et al. [30] and transported to the insectarium in labelled 750 mL mineral water bottles and raised at an ambient temperature of 28 °C with 80% relative humidity and a 12-h light/dark cycle. Emerged adults were captured using a vacuum cleaner and placed at −20 °C for a few minutes to euthanize them. They were preserved in Eppendorf tubes filled with cotton and silica gel. Adult mosquitoes were identified morphologically at the species level using a stereo zoom binocular microscope according to a standard morphological key [31].

### 2.4. Characterization of Water Collections

For each water sample, general geographical and physical data were recorded, such as GPS location, type of water sampling, depth (≤0.5 m or >0.5 m), area size (≤5 m^2^ or >5 m^2^), distance from dwellings (≤10 m or >10 m), water transparency (clear or opaque), presence of vegetation, the origin of the site (natural or artificial), the type of water (permanent or temporary) and exposure to sunlight (shaded, semi-shaded or sunny). Chemical characteristics including pH, temperature (°C), conductivity (μS/cm), salinity (g/L), and turbidity (in Formazin nephelometric units), were measured in the field using a portable HANNA HI (98,195) device (imLab, Wasquehal, France), in accordance with the protocol described by Nebbak et al. [32].

### 2.5. DNA Extraction and Molecular Identification of Anopheles Species

A sample of 117 *Anopheles* mosquitoes from different breeding sites was molecularly identified. The *Anopheles* specimens were homogenized in microtubes with stainless steel beads and lysis buffer using a TissueLyser II (Qiagen, Les Ulis, France). After incubating at 70 °C for one hour, the DNA was extracted using the NucleoSpin^®^ 96 Tissue Core Kit (Macherey-Nagel, Oensingen, Switzerland), following the manufacturer’s protocol. For each reaction, 3 µL of eluted template DNA was added to the PCR master mix, which contained DreamTaq polymerase, buffer, 2 mM MgCl_2_, and deoxyribonucleoside triphosphates (dNTPs) (ThermoFisher DreamTaq™ Green PCR Master Mix, ThermoFisher Scientific, Illkirch, France). The T1 Biometra thermocycler (Thermo Fisher Scientific, Illkirch, France) was programmed as follows: an initial step at 95 °C for 5 min, followed by 35 cycles of 95 °C for 1 min, 50 °C (annealing temperature) for 1 min, and 72 °C for 1 min, followed by a final extension step at 72 °C for 10 min. The quality of the PCR products was verified by agarose gel electrophoresis and visualized under ultraviolet light. The expected band size was 710 bp. The sequences were analyzed using Geneious Prime software version 2022.2.2. Next, the multiplex PCR protocol developed by Scott et al. was used to identify the sibling species of *Anopheles gambiae* [33], under the same conditions as the first PCR.

### 2.6. Statistical Analysis

The analyses were performed using R software, version 4.4.2 [34]. To ensure balance between groups, categorical variables such as pH, temperature, and depth were carefully dichotomized using specific cut-offs corresponding to their median (e.g., pH 8.3, temperature 29.82 °C, and depth 0.5 m, respectively). First, a descriptive analysis of the independent variables was performed. Then, each variable was individually integrated into a logistic regression model for univariate analysis. Variables with an effect and a *p*-value less than 0.25 were selected for the multivariate analysis as previously reported [35]. Larval positivity in water samples was modeled according to the characteristics of each sample using a random-effects logistic regression model that took into account the spatialization of the data via the water collection as a random effect. Additionally, larval density was assessed for a standardized volume of one liter of water. When it was not possible to collect one liter directly, density was estimated by proportional extrapolation from the actual volume collected. For statistical analysis, larval density was defined as the average value of the densities measured during the various studies for each habitat. larval density at breeding sites was analyzed as a dependent variable using negative binomial regression with the breeding site as a random effect. The significant variables (*p* < 0.05) and their interactions were selected using a stepwise selection procedure based on minimization of the Akaike information criterion (AIC) to construct the final model.

## 3. Results

### 3.1. Description of Anopheles Breeding Sites

In Nouakchott, most of the 60 water collections studied were temporary (47 out of 60, or 78.3%), while 13 (21.6%) were permanent. Of these, 54 (90%) were artificial and only six (10%) were natural. These collections were spread across several neighborhoods in the city and were mainly located in the most densely populated residential areas, particularly in Teyaret, Dar Naim, Sebkha, Tevragh-Zeina, El Mina and Riyadh. The surveys were conducted between May 2023 and April 2024, and the geographical location of the sampling sites is shown in Figure 1. Of the 60 water collections visited, 20 (33.3%) were positive for immature stages of *Anopheles* (Figure 1, Table S1). The sources included agricultural wastewater puddles (n = 4, 20%), water tanks (n = 2, 10%), barrels (n = 2, 10%), groundwater (n = 2, 10%), rainwater (n = 2, 10%), drains and pits (n = 2, 10%). Other sources, each representing 5%, included stagnant rainwater and groundwater (n = 1), a pit (n = 1), a water storage pond (n = 1), a fountain bollard drain (n = 1), a pipe leak (n = 1) and well water storage (n = 1). Figure S1 shows the main breeding sites of *Anopheles* spp. Table 1 summarizes their chemical characteristics, and Table S2 details the distribution of the 294 observations according to the type of water sampling type, thus providing an overview of the conditions favorable to the presence of larvae.

### 3.2. Factors Associated with the Positivity of Water Collections for Anopheles Larvae

Univariate logistic regression with random effect analysis of water collections positivity for *Anopheles* larvae was performed to identify the main factors associated with larval positivity in the examined water collections (Table S3). Then a multivariate logistic regression analysis with random effects revealed that the presence of *Culex* and *Aedes*, as well as pH and temperature, were statistically significant and independently associated with water collections positivity for *Anopheles* (Table 2). The presence of *Aedes* larvae was a protective factor (aOR 0.18, 95%CI [0.05–0.60], *p*-value = 0.006). Risk factors were the presence of *Culex* larvae (aOR 3.03, 95%CI [1.14–8.07], *p*-value = 0.006), a pH ≥ 8.3 (aOR 3.17, 95%CI [1.32–7.61], *p*-value = 0.010) and a temperature >29.82 °C (aOR 5.95, 95%CI [2.09–16.92], *p*-value < 0.001).

### 3.3. Factors Associated with the Density of Anopheles Larvae at Breeding Sites

A univariate binomial negative regression with random effect analysis of the number of *Anopheles* larvae at breeding sites was performed to identify the main factors associated with the density of *Anopheles* larvae (Table S4). Risk factor was a temperature >29.82 °C (cOR 2.88, 95%CI [1.25–6.63], *p*-value < 0.013) and protective factor was a depth >0.5 m (cOR 0.34, 95%CI [0.15–0.77], *p*-value = 0.009).

No model could be obtained using multivariate negative binomial regression with random effects analysis of the density of *Anopheles* larvae at breeding sites.

### 3.4. Species Composition of Anopheles

Of 117 *Anopheles* mosquitoes sampled from various breeding sites, 116 were *An. arabiensis* and one was *An. multicolor* (in breeding site G4, Figure 1).

## 4. Discussion

The impact of malaria is strongly influenced by environmental factors, among which the physicochemical parameters of mosquito breeding sites play a central role, significantly affecting density, survival, distribution and capacity of vector populations [36,37,38]. In this context, controlling *Anopheles* larvae is one of the most effective strategies for preventing malaria transmission in Nouakchott. This is particularly relevant given that recent studies highlight the resistance of *Anopheles* mosquitoes to pyrethroids [14], which are commonly used to treat mosquito nets. Therefore, implementing such larval control program effectively requires a thorough understanding of their larval habitat characteristics.

The present study demonstrates the persistence of *Anopheles* mosquito larvae in Nouakchott. Artificial sites were the most common type of *Anopheles* breeding site. This finding is consistent with the results of previous studies in Nouakchott [26], as well as in other countries in sub-Saharan Africa such as Sudan [39], Ghana [40]. However, in studies conducted in Burkina Faso [41], and Nigeria [42], *An. gambiae* larvae were mainly found in natural sites. In fact, puddles were the most common artificial breeding sites, as has already been reported in other studies conducted in urban areas in Benin [24]. Agricultural puddles and stagnant water puddles were also encountered, as in a study on mosquito abundance and physicochemical characteristics of their breeding sites conducted in Egypt, where *Anopheles* was encountered [19]. It also appears that groundwater promotes the proliferation of *Anopheles* mosquitoes, with three breeding sites found to have originated from rising groundwater levels. These results are not generally found in several studies of the sub-regions [43,44]. Some rainwater sources had relatively high salinity or conductivity, which could be explained by mixing with saline groundwater or by the dissolution of salt deposits present on the surface. Furthermore, Nouakchott’s low altitude, between 1 and 10 m, could also accentuate the influence of coastal waters on rainwater.

In our study, among 50 observations of *Culex* larvae in water collections, 29 coexisted with the presence of *Anopheles* larvae. This finding is consistent with a study conducted in Tanzania [45]. Another study conducted in Kenya revealed that *An. arabiensis* and *Culex quinquefasciatus* larvae shared the same aquatic habitats with coexistence rates higher than those expected by chance [46]. Among the species that pose a significant threat to public health, during our study, we observed in five observations the coexistence of *Ae. aegypti* and *Anopheles* larvae at water collections. *Aedes aegypti* has been found to coexist with *Anopheles* in the same larval habitats, as demonstrated in previous studies [24,47,48]. A study conducted in Nigeria revealed that the most species cohabitated in metal cans and clay pots were *Ae. aegypti*, *An. gambiae*, and *An. funestus* [48]. Additionally a study conducted in Kinshasa, Democratic Republic of Congo, revealed similar trends, showing the coexistence of *Aedes* and *Anopheles* species at breeding sites [49]. On the contrary, in Zanzibar, *Anopheles* was rarely found cohabiting with *Culex* and *Aedes* in the same breeding sites [50]. Semi-natural experiments conducted in Tanzania demonstrated that when *Ae. aegypti* larvae shared containers with *Anopheles* species (*An. arabiensis*, *An. gambiae sensu stricto*, and *An. funestus*), the *Anopheles* larvae exhibited reduced survival, slower development, and smaller adult size. In some cases, larval disappearance was observed, suggesting possible predation by *Ae. aegypti* [45]. In Nouakchott, the positive association observed between *Anopheles* and *Culex* larvae, as well as the negative association with *Ae. aegypti*, could be explained by differential habitat preferences and local ecological interactions. For example, *Anopheles* and *Culex* may share similar breeding sites that are favorable to their development such as shallow, stagnant water with certain physicochemical characteristics, while *Ae. aegypti*, often associated with smaller, less permanent artificial containers, find these same sites less suitable. Competition for resources or predation of larvae could also influence the co-occurrence of species.

The presence of *Anopheles* larvae in the city of Nouakchott was positively associated with pH, the majority of *Anopheles* breeding sites had a pH ≥ 8.3. This appears to be similar to the findings of a study conducted in Ouagadougou, where *An. funestus* preferred high pH environments [41]. A study conducted in Ethiopia also indicates that pH is associated with an increase in the density of *Anopheles* larvae [51]. An alkaline pH can promote microbial and algal growth, thereby increasing food availability for *Anopheles* larvae.

The majority of *Anopheles* larval development sites exhibit water temperature > 29.82 °C. Indeed, results of the present study, showed that presence of *Anopheles* larvae is positively correlated with temperature, this is consistent with the findings of Kabore et al. from Burkina Faso [41]. High temperatures probably accelerate larval development and shorten the mosquito life cycle, thereby increasing their abundance in warm habitats. However, Dejenie et al. demonstrated that the abundance of *Anopheles* larvae was negatively correlated with temperature [51].

Our study also documented the presence of *An. arabiensis* and *An. multicolor* in the city of Nouakchott, as shown in previous studies [14,52]. *Anopheles multicolor* was first reported by Lemrabott et al. [52] in highly saline breeding sites in Nouakchott, during a study on the salinity tolerance of *An. arabiensis* larvae in Nouakchott, Mauritania.

This study has certain limitations. First, it was conducted over a period of one year, which means that long-term climatic variations such as El Niño or La Niña could not be taken into account. In addition, certain environmental factors were not directly assessed, including the presence of predators and the concentration of organic nutrients; nevertheless, the *Anopheles* breeding sites were mainly artificial (75%) and therefore independent of these climatic phenomena. Thirdly, in some cases, residents refused access to their homes after an initial visit, which limited subsequent follow-up. In addition, some water collections dried up, reducing the number of monitoring sessions. Finaly, no multivariate negative binomial model could be obtained for larval density due to sample size limitations. These density analyses are therefore only exploratory, and more data are needed to determine which type of breeding sites are most productive for *Anopheles* larvae.

## 5. Conclusions

This study aimed to describe the physicochemical characteristics of *Anopheles* breeding sites in the urban area of Nouakchott, Mauritania. These breeding sites are primarily artificial, including puddles, agricultural puddles, stagnant puddles, and groundwater. As *Anopheles* mosquitoes are still present and malaria remains one of the main public health problems in Mauritania as a whole, and as *Anopheles* resistance to pyrethroids has already been proven, rapid measures are needed to manage *Anopheles* populations. Identifying “high-risk” habitats (artificial, shallow, warm, and alkaline water bodies) enables urban malaria programs to prioritize interventions, such as environmental management (reduction at source) and the targeted application of biological larvicides at specific times of the year.

## Figures and Tables

**Table 1:** Chemical characteristics of *Anopheles* spp. breeding sites.

Breeding Site	N ^1^	Type of Water Collection	pH ^2^	Salinity ^2^	Turbidity ^2^	Temperature ^2,^*	Conductivity ^2^
G1	12	Well water storage	8.6, 0.2	0.2, 0.03	219, 33	27.7, 0.8	425, 75
G2	8	Stagnant rainwater and groundwater	8.3, 0.1	0.9, 0.33	920, 361	27, 0.9	1700, 629
G3	5	Groundwater	8.7, 0.2	2.3, 0.62	2039, 536	28.1, 1.2	4089, 1 070
G4	3	Groundwater	8.2, 0.5	5.0, 1.3	4380, 1104	35.9, 2.92	8737, 2202
G5	2	Water tank	7.7, 0.04	0.1, 0.05	38, 25.50	31.9, 8.1	88, 64.03
G7	14	agricultural wastewater puddle	8.3, 0.3	0.98, 0.07	902, 61	27.8, 1.2	1810, 122
G8	14	agricultural wastewater puddle	8.2, 0.13	0.9, 0.92	848, 55.7	29.5, 1.20	1700, 108
G9	11	agricultural wastewater puddle	8.4, 0.19	2.2, 0.43	1866, 361	29.9, 1.15	3729, 717
G10	7	agricultural wastewater puddle	8.2, 0.2	0.68, 0.17	642, 157	33.0, 0.6	1283, 313
G15	7	Pipe leak	8.3, 0.2	0.23, 0.07	222, 70	31.1, 1.6	443, 139
G18	10	Water storage pond	8.3, 0.11	0.5, 0.12	545, 109	30, 1.03	1091, 207
G19	6	Fountain bollard drain	7.9, 0.2	0.66, 0.09	632, 73	32.2, 1.2	1266, 147
G20	9	Drain and pit	8.2, 0.13	0.5, 0.1	542, 97	31.0, 1.4	984, 138
G21	8	Drain and pit	8.2, 0.2	1.0, 0.08	900, 68	34.3, 1.3	1813, 138
G27	9	Pit	8.6, 0.2	1.3, 0.4	1097, 357	29.6, 1.7	2191, 708
G28	5	Water tank	9.0, 0.1	0.1, 0.02	101, 17.5	31.4, 0.6	203, 35
G37	1	Rainwater	7.8, ND	4.3, ND	3654, ND	32.8, ND	7307, ND
G39	2	Rainwater	8.0, 0.4	1.6, 1.6	1324, 1259	32, 4	2664, 2540
G44	6	Barrel	8.0, 0.3	0.10, 0.02	94, 15	27.4, 1.5	188, 30
G47	5	Barrel	7.9, 0.2	0.10, 0.02	102, 17	31.6, 0.5	187, 42

^1^ Number of observations; ^2^ Mean and standard error; * Degree Celsius; ND: not determined (the standard error of the mean cannot be calculated for a single value).

**Table 2:** Multivariate logistic regression with random effect analysis of water collections positivity for *Anopheles* larvae.

		N	P	aOR	95%CI	*p*-Value
***Culex***	No	189	21	1	1.14–8.07	0.026
	Yes	105	29	3.03		
***Aedes***	No	178	45	1	0.05–0.60	0.006
	Yes	116	5	0.18		
**pH**	<8.3	148	12	1	1.32–7.61	0.010
	≥8.3	146	38	3.17		
**Temperature (°C)**	≤29.82	147	13	1	2.09–16.92	< 0.001
	>29.82	147	37	5.95		

N = Number of observations; P = Number of positive observations to *Anopheles* larvae; aOR = adjusted Odds ratio; 95%CI = 95% Confidence interval of aOR; pH and temperature cutoff values correspond to the median of their data distribution.

## Data Availability

The data presented in this study are available on request from the corresponding author.

## Supplementary Materials

The following supporting information can be downloaded at: https://www.mdpi.com/article/10.3390/tropicalmed11020042/s1, Supplementary Figure S1: Photographs of the main *Anopheles* spp. breeding sites in Nouakchott; Table S1: Geographical location of *Anopheles* spp. breeding sites in the three Wilaya of Nouakchott; Table S2: Number of observations per water collection; Table S3: Univariate logistic regression with random effect analysis of water collections positivity for *Anopheles* spp. larvae.; Table S4: Univariate binomial negative regression with random effect analysis of number of *Anopheles* spp. larvae at breeding sites.

## Abbreviations

The following abbreviations are used in this manuscript:

aOR	Adjusted odd ratio
cOR	Crude odd ratio
CI	Confidence interval
