# Supplementary material for: Physicochemical Properties of Anopheles Mosquito Larval Habitats in Nouakchott, Mauritania
Source: Trop Med Infect Dis. 2026 Feb 3;11(2):42. doi: 10.3390/tropicalmed11020042 (PMC12945047; doi:10.3390/tropicalmed11020042)
Supplement: Supplementary file 1 [file tropicalmed-11-00042-s001.zip › Table S1.pdf]

Table S1. Geographical location of *Anopheles* spp. breeding sites in the three Wilaya of Nouakchott.

Breeding site N°	Wilaya	Moughataa	Type of water collection	Latitude	Longitude
G1	NN	Teyaret	Well water storage	18.12928 N	15.93395 W
G2	NN	Teyaret	Stagnant rainwater and groundwater	18.12949 N	15.93400 W
G3	NN	Teyaret	Groundwater	18.12930 N	15.93408 W
G4	NN	Teyaret	Groundwater	18.12933 N	15.93403 W
G5	NN	Dar Naim	Water tank	18.11865 N	15.92471 W
G7	WN	Sebkha	agricultural wastewater puddle	18.07981 N	15.98816 W
G8	WN	Sebkha	agricultural wastewater puddle	18.08095 N	15.98543 W
G9	WN	Sebkha	agricultural wastewater puddle	18.08111 N	15.98466 W
G10	WN	Sebkha	agricultural wastewater puddle	18.08093 N	15.98561 W
G15	WN	Tevragh-Zeina	Pipe leak	18.16604 N	15.99680 W
G18	WN	Sebkha	Water storage pond	18.07517 N	15.99706 W
G19	WN	Sebkha	Fountain bollard drain	18.07785 N	15.99379 W
G20	WN	Sebkha	Drain and pit	18.07796 N	15.98729 W
G21	SN	El Mina	Drain and pit	18.06508 N	15.98855 W
G27	SN	El Mina	pit	18.06979 N	15.99166 W
G28	SN	Riyadh	Water tank	18.00256 N	15.93414 W
G37	NN	Teyaret	Rainwater	18.12784 N	15.93338 W
G39	NN	Teyaret	Rainwater	18.12544 N	15.93496 W
G44	WN	Tevragh-Zeina	Barrel	18.08515 N	15.99129 W
G47	WN	Tevragh-Zeina	Barrel	18.09882 N	15.98465 W

NN: North Nouakchott; WN: West Nouakchott; SN: South Nouakchott.
