# Supplementary material for: Physicochemical Properties of Anopheles Mosquito Larval Habitats in Nouakchott, Mauritania
Source: Trop Med Infect Dis. 2026 Feb 3;11(2):42. doi: 10.3390/tropicalmed11020042 (PMC12945047; doi:10.3390/tropicalmed11020042)
Supplement: Supplementary file 1 [file tropicalmed-11-00042-s001.zip › Table S2.pdf]

Table S2. Number of observations per water collection.

Water collections	Number of observations
G1	12
G2	8
G3	5
G4	3
G5	2
G6	2
G7	14
G8	14
G9	11
G10	7
G11	9
G12	8
G13	3
G14	3
G15	7
G16	2
G17	2
G18	10
G19	6
G20	9
G21	8
G22	1
G23	13
G24	13
G25	1
G26	1
G27	9
G28	5
G29	2
G30	5
G31	1
G32	8
G33	2
G34	2
G35	7
G36	2
G37	1
G38	1
G39	2
G40	4
G41	4
G42	3
G43	10
G44	6
G45	1
G46	5
G47	5
G48	3
G49	1
G50	2
G51	1
G52	3
G53	7
G54	6
G55	2
G56	3
G57	3
G58	1
G59	1
G60	2
