# Supplementary material for: Physicochemical Properties of Anopheles Mosquito Larval Habitats in Nouakchott, Mauritania
Source: Trop Med Infect Dis. 2026 Feb 3;11(2):42. doi: 10.3390/tropicalmed11020042 (PMC12945047; doi:10.3390/tropicalmed11020042)
Supplement: Supplementary file 1 [file tropicalmed-11-00042-s001.zip › Table S3.pdf]

**Table S3.** Univariate logistic regression with random effect analysis of water collections positivity for *Anopheles* spp. larvae.

| | | N | P | cOR | 95%CI | p-value |
|---|---|---|---|---|---|---|
| **Presence of *Culex* larvae** | | | | | | |
| | No | 189 | 21 | 1 | | |
| | Yes | 105 | 29 | 3.06 | 1.28 – 7.31 | 0.012 |
| **Presence of *Aedes* larvae** | | | | | | |
| | No | 178 | 45 | 1 | | |
| | Yes | 116 | 5 | 0.16 | 0.05– 0.48 | 0.001 |
| **pH** | | | | | | |
| | < 8.3 | 148 | 12 | 1 | | |
| | ≥ 8.3 | 146 | 38 | 3.77 | 1.69 – 8.41 | 0.001 |
| **Salinity (g/L)** | | | | | | |
| | < 0.18 | 146 | 14 | 1 | | |
| | ≥ 0.18 | 148 | 36 | 2.38 | 0.77 – 7.36 | 0.068 |
| **Turbidity (ppm)** | | | | | | |
| | < 152 | 147 | 13 | 1 | | |
| | ≥ 152 | 147 | 37 | 2.91 | 1.15 – 7.39 | 0.024 |
| **Temperature (°C)** | | | | | | |
| | ≤ 29.82 | 147 | 13 | 1 | | |
| | > 29.82 | 147 | 37 | 7.56 | 2.77 – 20.65 | < 0.001 |
| **Conductivity (µs/cm)** | | | | | | |
| | < 303 | 147 | 14 | 1 | | |
| | ≥ 303 | 147 | 36 | 2.48 | 0.99– 6.22 | 0.052 |
| **Depth (m)** | | | | | | |
| | ≤ 0.5 | 94 | 21 | 1 | | |
| | > 0.5 | 200 | 29 | 0.45 | 0.18 – 1.14 | 0.091 |
| **Size (m²)** | | | | | | |
| | ≤ 5 | 73 | 16 | 1 | | |
| | > 5 | 221 | 34 | 0.68 | 0.19– 2.46 | 0.560 |
| **Distance to Habitat (m)** | | | | | | |
| | ≤ 10 | 217 | 35 | 1 | | |
| | > 10 | 77 | 15 | 1.58 | 0.37 – 6.69 | 0.53 |
| **Water collection type** | | | | | | |
| | Natural | 20 | 5 | 1 | | |
| | Artificial | 274 | 45 | 0.24 | 0.03 – 1.93 | 0.181 |
| **Water collection state** | | | | | | |
| | Permanent | 77 | 17 | 1 | | |
| | Temporary | 217 | 33 | 0.34 | 0.08 – 1.52 | 0.160 |
| **Exposure to the sun** | | | | | | |
| | Shaded | 52 | 5 | 1 | | |
| | semi shaded | 136 | 19 | 1.42 | 0.24 – 8.54 | 0.703 |
| | Sunny | 106 | 26 | 4.35 | 0.72 – 26.40 | 0.110 |
| **Water colour** | | | | | | |
| | Clear | 193 | 27 | 1 | | |
| | Dark | 101 | 23 | 3.12 | 0.78 – 12.54 | 0.11 |
| **Plants presence** | | | | | | |
| | No | 206 | 32 | 1 | | |
| | Yes | 88 | 18 | 1.45 | 0.37 – 5.60 | 0.591 |

N = Number of observations; P = Number of positive observations for *Anopheles* larvae; cOR = crude Odd ratio; 95%CI = 95% Confidence interval of cOR.
