# Supplementary material for: Physicochemical Properties of Anopheles Mosquito Larval Habitats in Nouakchott, Mauritania
Source: Trop Med Infect Dis. 2026 Feb 3;11(2):42. doi: 10.3390/tropicalmed11020042 (PMC12945047; doi:10.3390/tropicalmed11020042)
Supplement: Supplementary file 1 [file tropicalmed-11-00042-s001.zip › Table S4.pdf]

Table S4. Univariate binomial negative regression with random effect analysis of number of *Anopheles* larvae at breeding sites.

		N	cOR	95%CI	p-value
**pH**					
	< 8.3	6	1		
	≥ 8.3	12	0.60	0.22 – 1.65	0.330
**Salinity (g/L)**					
	< 0.18	6	1		
	≥ 0.18	12	0.76	0.26 – 2.19	0.600
**Turbidity (ppm)**					
	< 152	6	1		
	≥ 152	12	0.76	0.26 – 2.19	0.600
**Temperature (°C)**					
	≤ 29.82	7	1		
	> 29.82	11	2.88	1.25– 6.63	0.013
**Conductivity (µs/cm)**					
	< 303	7	1		
	≥ 303	11	0.79	0.28 – 2.25	0.660
**Depth (m)**					
	≤ 0.5	10	1		
	> 0.5	8	0.34	0.15 – 0.77	0.009
**Size (m^2)**					
	≤ 5	6	1		
	> 5	12	0.81	0.27 – 2.46	0.710
**Distance to Habitat (m)**					
	≤ 10	16	1		
	> 10	2	0.30	0.07 – 1.31	0.110
**Water collection type**					
	Natural	2	1		
	Artificial	16	1.28	0.28 – 5.84	0.750
**Water collection state**					
	Permanent	3	1		
	Temporary	15	1.57	0.44 – 5.65	0.490
**Exposure to the sun**					
	Shaded/semi shaded	8	1		
	Sunny	10	1.09	0.37 – 3.18	0.880
**Water color**					
	Clear	15	1		
	Dark	3	2.95	0.97 – 9.01	0.057

N = Number of observations; cOR = crude Odd ratio; 95%CI = 95% Confidence interval of cOR.
