# Supplementary figures and images for: Physicochemical Properties of Anopheles Mosquito Larval Habitats in Nouakchott, Mauritania
Source: Trop Med Infect Dis. 2026 Feb 3;11(2):42. doi: 10.3390/tropicalmed11020042 (PMC12945047; doi:10.3390/tropicalmed11020042)

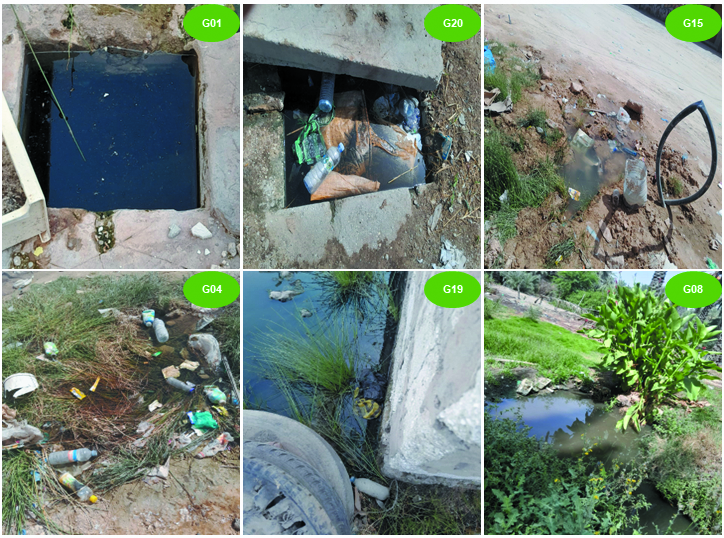

Supplement: Supplementary file 1 [file tropicalmed-11-00042-s001.zip › Supplementary Figure S1.tif]
